# Nicotine and fluoxetine alter adolescent dopamine-mediated behaviors via 5-HT_1A_ receptor activation

**DOI:** 10.3389/fpsyt.2024.1380123

**Published:** 2024-06-11

**Authors:** Menglu Yuan, Frances M. Leslie

**Affiliations:** Department of Pharmaceutical Sciences, School of Pharmacy and Pharmaceutical Sciences, University of California Irvine (UCI), Irvine, CA, United States

**Keywords:** adolescence, age-dependent, nicotine, fluoxetine, cocaine, behavioral acquisition, self-administration

## Abstract

**Introduction:**

Abuse or misuse of tobacco, e-cigarettes, or antidepressants may have serious clinical consequences during adolescence, a sensitive period during brain development when the distinct neurobiology of adolescent serotonin (5-HT) and dopamine (DA) systems create unique behavioral vulnerabilities to drugs of abuse.

**Methods:**

Using a pharmacological approach, we modeled the behavioral and neurochemical effects of subchronic (4-day) nicotine (60µg/kg, i.v.) or fluoxetine (1mg/kg, i.v.) exposure in adolescent and adult male rats.

**Results:**

Nicotine and fluoxetine significantly enhance quinpirole-induced locomotor activity and initial cocaine self-administration in adolescents, but not adults. These effects were blocked by serotonin 5-HT_1A_ receptor antagonists, WAY-100,635 (100 µg/kg, i.v.) or S-15535 (300 µg/kg, i.v.). Neurochemical and anatomical autoradiographic analysis of 8-OH-DPAT-stimulated [^35^S]GTPγS reveal that prior exposure to nicotine and fluoxetine results in both overlapping and distinct effects on regional 5-HT1A receptor activity. Both fluoxetine and nicotine enhance adolescent 5-HT1A receptor activity in the primary motor cortex (M1), whereas fluoxetine alone targets prefrontal cortical neurocircuitry and nicotine alone targets the amygdala.

**Discussion:**

Given their different pharmacological profiles, comparison between WAY-100,635 and S-15535 indicates that postsynaptic 5-HT_1A_ receptors mediate the behavioral effects of prior nicotine and fluoxetine exposure. In addition, within the adolescent M1, maladaptive changes in 5-HT signaling and 5-HT_1A_ activity after nicotine or fluoxetine exposure may potentiate hyper-responsiveness to dopaminergic drugs and prime adolescent vulnerability for future substance abuse.

## 1 Introduction

Adolescence is a period of brain development during which neurotransmitter systems are actively maturing, with dopamine (DA) exhibiting particularly dynamic changes (1–3). These neurochemical changes not only underlie executive, cognitive, and emotional maturation, but also mark adolescence as a vulnerable transition period when the initiation of substance abuse and onset of psychiatric disorders typically emerge (4, 5).

Among teenagers, recreational use of electronic nicotine delivery systems (e-cigarettes) has escalated exponentially (6, 7). Originally marketed as a smoking cessation aid, recreational e-cigarette use, or vaping, is currently more prevalent than combustible cigarette use among young people (8). Furthermore, teenagers who vape are more likely to smoke, and e-cigarettes may be a “gateway” to future tobacco abuse (9). Consequently, the emerging popularity of e-cigarettes poses an urgent threat for adolescent health, as tobacco remains the leading cause of disease and death worldwide and the negative health effects of e-cigarette use has yet to be fully understood.

Initiation of smoking typically occurs during adolescence, with approximately 90% of adult smokers starting before age 18 (10). Furthermore, adolescent nicotine and tobacco use are associated with disproportionately higher rates of future substance abuse (11–14) and depression compared to their nonsmoking peers (15). To treat teen depression, fluoxetine (Prozac), a selective serotonin reuptake inhibitor (SSRI), is commonly prescribed as an antidepressant (16). Although beneficial, SSRIs can increase suicidal thoughts and behaviors, agitation, anxiety, and lead to further depression in teenagers (17–19).

Adolescent rodents, conservatively defined as between postnatal day (P) 28 and 42 (20), demonstrate unique responses to both SSRIs and nicotine. Chronic SSRI treatment upregulates hippocampal neurotrophic factors in adult rats, but pro-apoptotic factors in adolescents (21). Fluoxetine also enhances cell proliferation in adult rats, with little change in adolescents (22). Compared to adults, adolescent rats associate a greater rewarding effect with nicotine in conditioned place preference and self-administration studies (23–28). In contrast, adolescents display both less aversion (25, 28, 29) and blunted withdrawal symptoms to nicotine compared to adults (30, 31). Nicotine induces inflammatory markers in adolescent brain, in contrast to the anti-inflammatory effect observed in adults (32). Nicotine also exerts unique effects on adolescent DA and 5-HT systems; specifically, receptor activation of both DA and 5-HT receptor subtypes underlies nicotine-induced increases of drug reinforcement in adolescents, but not adults (32, 33).

In adolescent rodents, but not adults, brief exposure to nicotine enhances cocaine locomotor sensitization, conditioned place preference, and self-administration as well as quinpirole-induced activity (33–37). Not only does nicotine selectively increase 5-HT content and transporter binding in adolescent forebrain regions, but several unique behavioral effects of adolescent nicotine exposure are blocked by co-administration of a 5-HT_1A_ receptor (5-HT_1A_R) antagonist (33). Together, these findings suggest that age-specific alterations in 5-HT and 5-HT_1A_R signaling during adolescence may underlie enhanced behavioral sensitivity to DA drugs after nicotine exposure.

We have now used a behavioral pharmacology approach to test whether direct changes to endogenous 5-HT signaling with fluoxetine in adolescence can also potentiate DA-mediated behaviors, including cocaine self-administration and ambulatory activity induced by quinpirole, a D_2_ receptor agonist. In addition, we have characterized the possible role of 5-HT_1A_Rs, using the antagonist WAY-100,635 and the partial agonist S-15535, in the effects of nicotine and fluoxetine on the adolescent brain. Furthermore, through [^35^S]GTPγS binding, an *in vitro* approach to examine receptor-G protein coupling, we have shown unique effects of adolescent fluoxetine and nicotine exposure on 5-HT_1A_R function.

## 2 Materials and methods

### 2.1 Animals

Male Sprague-Dawley rats (Charles River Labs, Hollister, CA) arrived at P17 with dams or at P75. Rats were group-housed in an AAALAC-accredited vivarium on a 12 hr light/dark cycle with unlimited access to food and water. Pups were weaned on P21. All experiments were performed during the light cycle and carried out in accordance with the Institutional Animal Care and Use Committee at the University of California, Irvine.

### 2.2 Drugs

Fluoxetine HCl (Sigma, St. Louis, MO) was dissolved in sterile water. Nicotine hydrogen tartrate (Sigma, St. Louis, MO) was dissolved in sterile saline and adjusted to pH 7.2 – 7.4; doses were calculated as free base. Cocaine HCl (Sigma, St. Louis, MO) (–),-quinpirole HCl (Tocris Bioscience, Bristol, UK), and WAY-100,635 (Sigma, St. Louis, MO) were dissolved in sterile saline. S-15535 (Sigma, St. Louis, MO) was dissolved in sterile saline that was acidified with lactic acid, and pH was adjusted to as close to neutrality as possible (pH > 5.0). L-741,626 (Tocris Bioscience, Bristol, UK) was dissolved in 50% ethanol/saline. Propofol was purchased from Abbot Laboratories (Chicago, IL).

### 2.3 Catheter Implantation

Rats, aged P24 or P82, were anesthetized with equithesin (0.0035 ml/g) and surgically implanted with an indwelling catheter in their jugular vein (37). During 3 days of recovery, catheters were flushed daily with heparinized saline solution (1000 units heparin per 30 ml bacteriostatic saline) to maintain patency. Catheter patency was verified by testing with propofol (5 mg/kg, i.v.) after drug pretreatment, and only animals showing rapid anesthesia were included in analyses.

### 2.4 Pretreatment

Adolescent (P28–31) and adult (P86–89) rats received a drug pretreatment for 4 consecutive days (32). Daily intravenous injections of fluoxetine (1 mg/kg), WAY-100,635 (100 µg/kg), fluoxetine + WAY-100,635, nicotine (60 µg/kg), or saline were administered during adolescence or adulthood. Behavioral testing was conducted the following day on either P32 or P90 (**Supplementary Figure 1**).

### 2.5 Locomotor Behavior

One day after pretreatment, at P32 for adolescents and P90 for adults, locomotor activity was recorded in an open field chamber (43.2 × 43.2 × 30.5 cm^3^) by 16 photobeams along the sides of each wall (MED Associates, Inc., St. Albans, VT). Following 30 min of habituation in the test chamber, rats received an injection of either quinpirole (0.4 mg/kg, i.p.), a selective D_2_ and D_3_ receptor agonist, or vehicle and returned for 30 min monitoring of horizontal activity. For acute 5-HT_1A_R antagonist studies, rats were given a single infusion of WAY-100,635 (100 µg/kg, i.v.), S-15535 (300 µg/kg, i.v.), or vehicle 20 min prior to locomotor testing.

### 2.6 Cocaine Self-Administration

One day after pretreatment, at P32 for adolescents and P90 for adults, acquisition of cocaine self-administration was evaluated in an operant chamber containing two nose poke holes (MED Associates, Inc., St. Albans, VT). Rats self-administered cocaine (0.5 mg/kg, i.v., 20 µl, 1.1s) in a single 1 hr session on a fixed ratio 1 (FR1) schedule. During each infusion, the cue light above the reinforced hole was illuminated; afterward the house light shut off for a 20 sec timeout where responses were counted but had no effect. To control for nonspecific activity, responses for the non-reinforced hole were recorded but had no programmed consequences. For acute 5-HT_1A_R antagonist studies, rats were given a single infusion of WAY-100,635 (100 µg/kg, i.v.), L-741,626 (2 mg/kg, i.p.), or vehicle 20 min prior to self-administration testing.

### 2.7 Tissue Preparation

On P32 or P90, 24 hrs after the last pretreatment infusion, brains were collected, rapidly frozen, and stored at -70°C until processing. Coronal sections of 20 µm thickness were cut in a cryostat maintained at -20°C. Sections were mounted onto ice-cold, gelatin-coated glass slides (Fisher Scientific, Waltham, MA). Slides were air dried for 30 min at room temperature and processed for 8-OH-DPAT-stimulated [^35^S]GTPγS binding.

### 2.8 [^35^S]GTPγS Autoradiography

8-OH-DPAT-stimulated [^35^S]GTPγS (Perkin Elmer, Boston, MA) autoradiography was as described by Hensler et al. (2007) (38) with slight modification. Slide-mounted sections were equilibrated in assay buffer (50 mM HEPES; 3 mM magnesium perchlorate; 0.2 mM EGTA; 100 mM NaCl; and 0.2 mM dithiothreitol; pH 7.4) for 10 min at room temperature. Sections were pre-incubated in 5’-guanylate diphosphate (2 mM GDP) for 15 min at room temperature, and then incubated for 60 min in HEPES buffer containing GDP (2 mM) and [^35^S]GTPγS (40 pM), with or without 8-OH-DPAT (1 µM). Basal [^35^S]GTPγS binding was determined in the absence of 8-OH-DPAT. Nonspecific [^35^S]GTPγS binding was determined in the presence of unlabeled GTPγS (10 µM). The incubation was stopped by two 5 min washes in ice-cold HEPES buffer (50 mM, pH 7.4), followed by a brief rinse in ice-cold deionized water. Sections were air dried at room temperature for 1 hr and exposed to Kodak Biomax MR film for 48 hrs.

### 2.9 Anatomical Analysis

Digitized autoradiograms were quantified with a MicroComputer Imaging Device (MCID Imaging Research, St. Catherine, Ontario, Canada; **Supplementary Figure 2**). Autoradiograms of 8-OH-DPAT-stimulated [^35^S]GTPγS binding were simultaneously exposed with [^14^C] standards (39). Standard calibration curves were generated from [^14^C] standards of optical density against radioligand concentration (dpm/mg tissue) and used to transform regional density values into relative radioactivity measures. Nonspecific binding of [^35^S]GTPγS was subtracted from basal binding and from specific, agonist-stimulated, binding. Specific binding was expressed as percent change from basal. Areas that were analyzed included subregions of the cortex (prelimbic, infralimbic, ventrolateral orbital, cingulate, agranular insular, primary motor, and secondary motor); amygdala (basolateral, central, and medial); striatum (caudate putamen, nucleus accumbens shell and core); hypothalamus (paraventricular nucleus and lateral); dorsal and ventral hippocampus; dorsal and median raphe; bed nucleus of the stria terminalis; periventricular thalamus; and ventral tegmental area.

### 2.10 Sample size and randomization

No statistical methods were used to predetermine sample sizes. Sample sizes used were similar to those reported in previous publications (32–36). Analysis of digitized autoradiograms were performed blind.

### 2.11 Data Analysis

Locomotor activity data are expressed as mean ± SEM and were analyzed by 3-way ANOVA comparing Age × Pretreatment × Drug or 2-way ANOVA comparing Pretreatment × Drug. All significant main effects were further analyzed by one-way ANOVA with Bonferroni *post hoc* comparisons. For acquisition of cocaine or sucrose self-administration, reinforced (R) and non-reinforced (NR) response data are expressed as mean ± SEM and were analyzed by 2-way ANOVA for Pretreatment × R/NR Responses, with repeated measures on Responses. Significant effects of reinforcement were analyzed by Bonferroni-corrected paired t-tests for each pretreatment group. Anatomical data for each brain region are expressed as mean ± SEM and were analyzed separately using 2-way ANOVA comparing Pretreatment × Age. Any significant main effects were further compared using Dunnett’s *post hoc* test. All analyses were completed using SPSS Statistics software. Outliers (≥2 SD from group mean) were removed prior to analysis.

## 3 Results

### 3.1 Fluoxetine pretreatment increases adolescent quinpirole-induced locomotion via 5-HT_1A_ receptors

**Figure 1:**
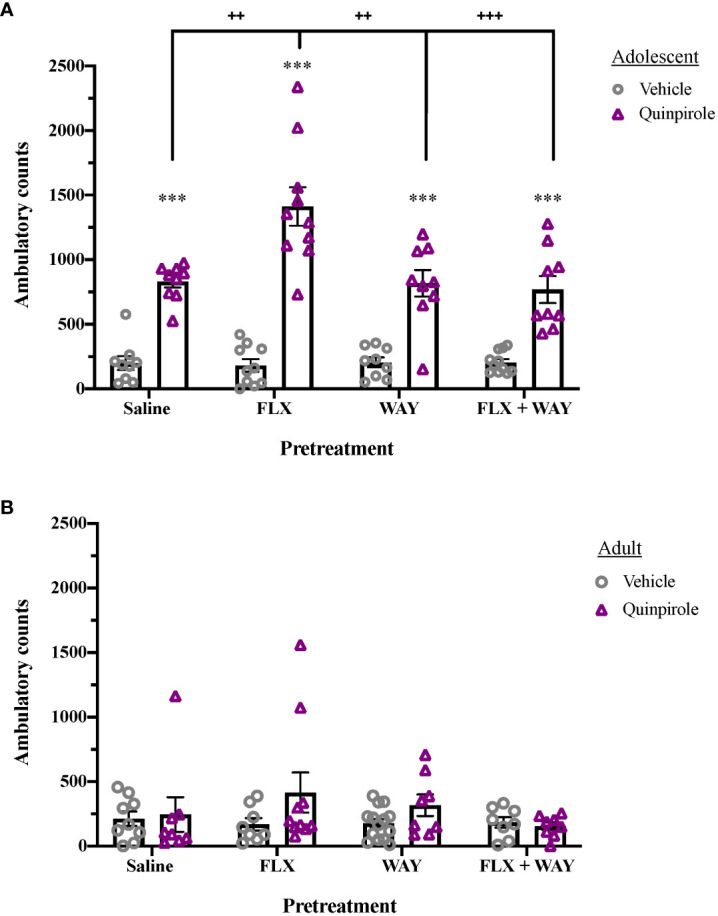
Age differences in the effect of fluoxetine and WAY-100,635 pretreatment on quinpirole-induced locomotion. **(A)** Quinpirole (0.4 mg/kg, i.p.) significantly induced locomotion in adolescents (***p < 0.001 vs. vehicle). Fluoxetine (1mg/kg, i.v.)-pretreated adolescents showed enhanced locomotor activity following administration of quinpirole (++p < 0.01 vs. saline), an effect that was blocked by pretreatment with WAY-100,635 (100 µg/kg, i.v.; +++p = 0.001 vs. FLX+WAY). WAY-100,635 pretreatment alone did not alter quinpirole-induced locomotor activity (n = 9 saline-vehicle, 9 saline-quinpriole, 10 FLX-vehicle, 10 FLX-quinpirole, 9 WAY-vehicle, 9 WAY-quinpirole, 11 FLX+WAY-vehicle, 9 FLX+WAY-quinpirole). **(B)** In adults, there were no significant effects of pretreatment or quinpirole on locomotor activity across groups (n = 9 saline-vehicle, 8 saline-quinpriole, 9 FLX-vehicle, 8 FLX-quinpirole, 8 WAY-vehicle, 8 WAY-quinpirole, 8 FLX+WAY-vehicle, 8 FLX+WAY-quinpirole). Data analyzed with analyzed by 3-way ANOVA, with Bonferroni *post hoc* tests. Bars represent mean ± SEM.

As shown in **Figure 1** fluoxetine pretreatment of adolescents, but not adults, increased quinpirole-induced locomotor activity via a 5-HT_1A_R mechanism. Overall effects of Age (F_1,126 = _96.189, p < 0.001), Pretreatment (F_3,126 = _3.255, p < 0.05), and Drug (F_1,126 = _111.913, p < 0.001) were seen, as well as significant interactions of Age × Pretreatment (F_3,126 = _4.696, p < 0.01), Age × Drug (F_1,126 = _88.538, p < 0.001), Pretreatment × Drug (F_3,126 = _4.301, p < 0.01], and Age × Pretreatment × Drug (F_3,126 = _4.937, p < 0.01). Quinpirole induced significant locomotor activity in adolescents (p < 0.001; **Figure 1A**), which was enhanced by fluoxetine pretreatment (p < 0.01). Co-administration of the 5-HT_1A_ receptor antagonist, WAY-100,635, during pretreatment blocked fluoxetine enhancement of quinpirole-induced locomotion in adolescents (p < 0.001). As has been shown previously (33), quinpirole did not induce significant locomotor activity in adults; nor did fluoxetine or WAY-100,635 pretreatment influence locomotor activity in this age group (**Figure 1B**).

### 3.2 Fluoxetine pretreatment enhances adolescent acquisition of cocaine self-administration via 5-HT_1A_ receptors

**Figure 2:**
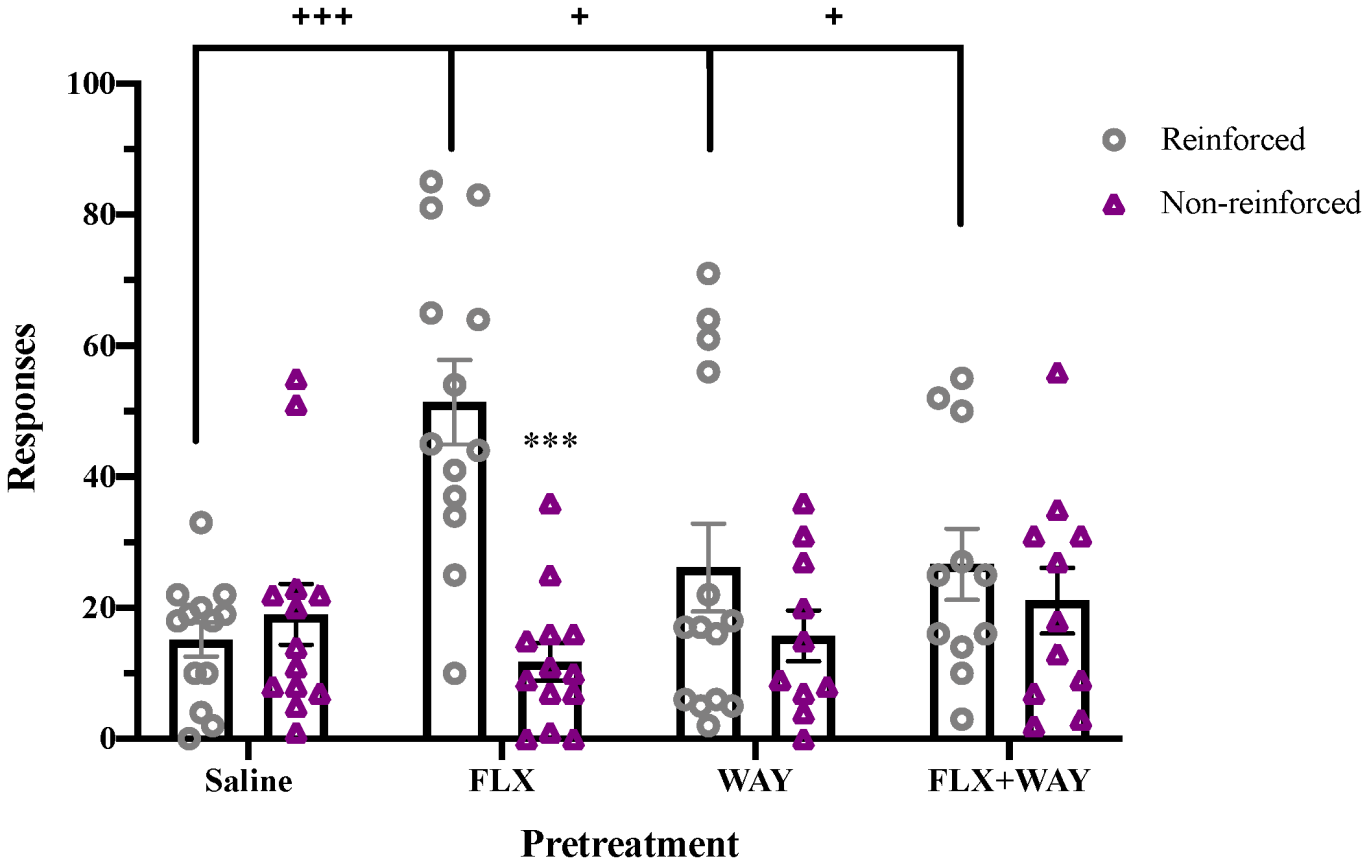
Fluoxetine pretreatment during adolescence enhanced cocaine self-administration, an effect blocked by co-administration of WAY-100,635. Fluoxetine (1mg/kg, i.v.)-pretreated adolescents had significantly higher reinforced responses for cocaine (0.5 mg/kg/inf, i.v.) compared to non-reinforced responses (***p < 0.001). Fluoxetine-pretreated adolescents also had greater total reinforced responses for cocaine compared to other pretreatment groups (+++p < 0.001 vs. saline, +p < 0.05 vs. WAY and FLX+WAY). WAY-100,635 (100 µg/kg, i.v.) alone did not alter cocaine self-administration. Data were analyzed by 2-way ANOVA, with repeated measures on Responses. Significant effects of reinforcement were analyzed by Bonferroni-corrected paired t-tests for each pretreatment group. Bars represent mean ± SEM. n = 13 saline, 14 FLX, 13 WAY, 11 FLX + WAY.

To examine the role of endogenous 5-HT in drug reinforcement, we evaluated the effect of fluoxetine pretreatment on subsequent cocaine self-administration in adolescents (**Figure 2**). A significant effect of Response (F_1,47 = _23.095, p < 0.001) and a Response x Pretreatment interaction (F_3,47 = _14.912, p < 0.001) were observed. Whereas saline-pretreated adolescent rats did not discriminate between reinforced and non-reinforced nosepokes in a novel self-administration chamber, those pretreated with fluoxetine exhibited significantly higher preference for the reinforced hole (p < 0.001) and higher reinforced responses than saline-pretreated controls (p < 0.001). Enhancement of cocaine reinforcement by fluoxetine treatment was blocked by co-administration of WAY-100,635, indicating that the effect was mediated by 5-HT_1A_ receptors. In contrast, fluoxetine pretreatment had no effect on initial cocaine self-administration in adults (**Supplementary Figure 3**).

### 3.3 Enhanced 5-HT_1A_R signaling persists after adolescent pretreatment with nicotine or fluoxetine

**Figure 3:**
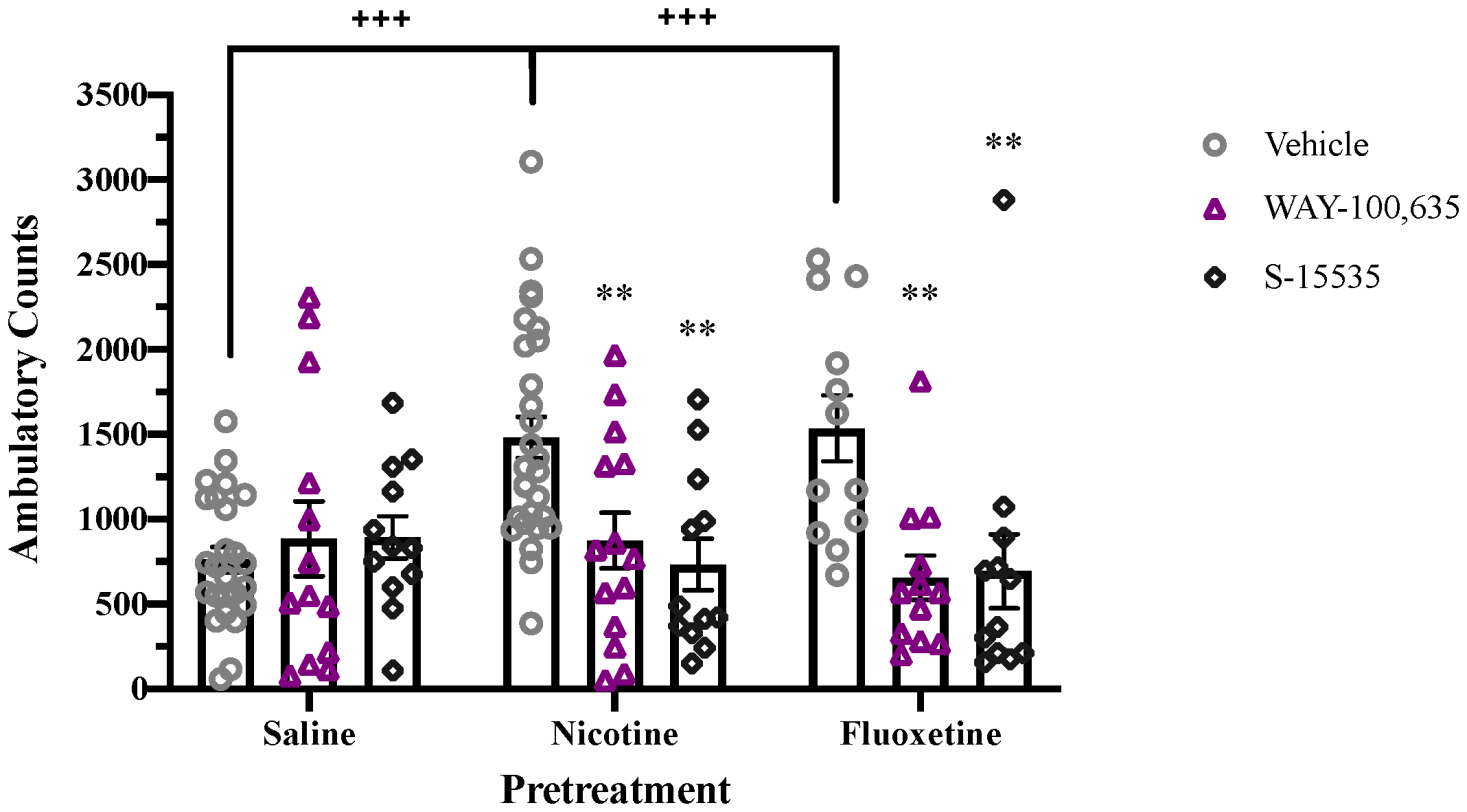
Acute 5-HT_1A_ receptor antagonism blocks enhancement of quinpirole-induced locomotion in nicotine- and fluoxetine-pretreated adolescent rats. Nicotine and fluoxetine pretreatment enhance quinpirole-induced locomotor activity (+++p < 0.001 vs. saline pretreatment). Acute administration of WAY-100,635 (100 µg/kg, i.v.) or S-15535 (300 µg/kg, i.v.) blocks the enhancement of quinpirole-induced locomotion in adolescents pretreated with nicotine (60 µg/kg, i.v.) or fluoxetine (1mg/kg, i.v.) (**p < 0.01 vs vehicle). Data analyzed with analyzed by 2-way ANOVA, with Bonferroni *post hoc* tests. Bars represent mean ± SEM. n = 25 saline-vehicle, 23 saline-WAY, 12 saline-S-15535, 28 nicotine-vehicle, 14 nicotine-WAY, 12 nicotine-S-15535, 12 fluoxetine-vehicle, 12 fluoxetine-WAY, 12 fluoxetine-S-15535.

Adolescent treatment with fluoxetine or nicotine induces persistent alteration in 5-HT_1A_ receptor signaling, even after drug pretreatment has ended. Following adolescent pretreatment with either drug, enhanced quinpirole-induced locomotor activity is observed which is blocked by acute administration of the 5-HT_1A_ receptor antagonist, WAY-100,635, or partial agonist, S-15535 (**Figure 3**). A significant overall effect of Antagonist (F_2,131 = _10.195, p < 0.001) and a Pretreatment x Antagonist interaction (F_4,131 = _4.488, p < 0.01) were observed. Pretreatment with nicotine or fluoxetine significantly enhanced quinpirole-induced locomotion in controls (p < 0.001), but not in animals treated acutely with WAY-100,635 or S-15535 (**Figure 3**).

**Figure 4:**
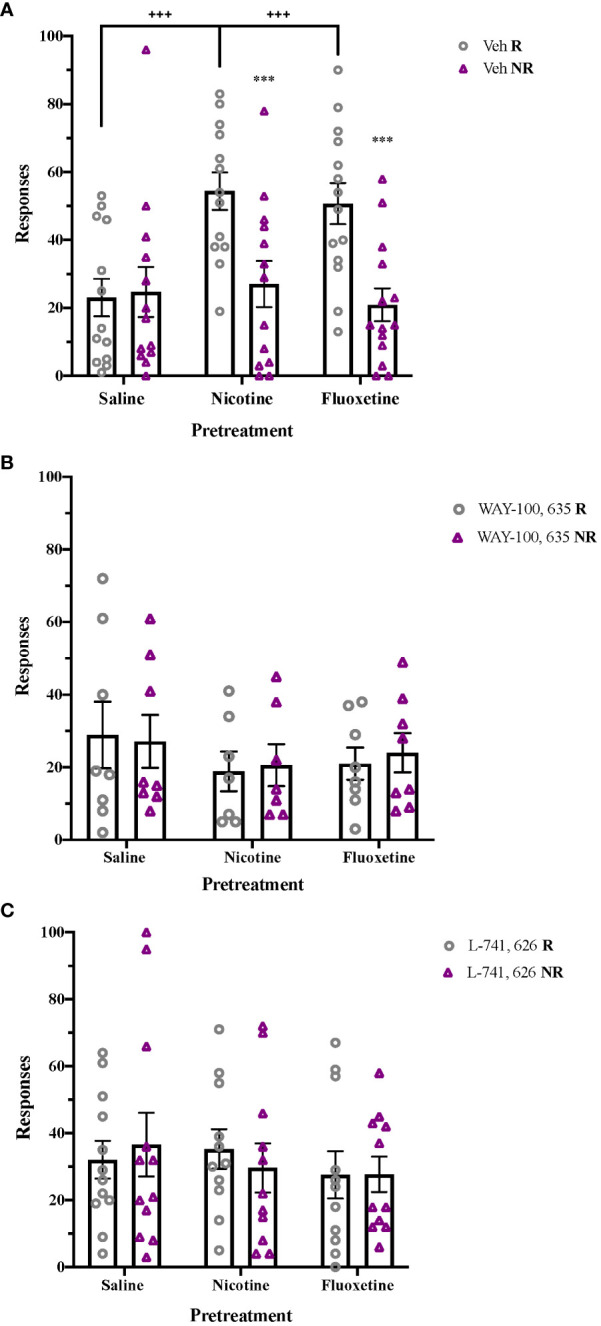
Post-treatment 5-HT_1A_ and D_2_ receptor activation mediate enhancement of cocaine self-administration after nicotine and fluoxetine pretreatment. **(A)** Adolescent rats pretreated with nicotine (60 µg/kg, i.v.) or fluoxetine (2 mg/kg, i.v.) had significantly greater reinforced responses compared to non-reinforced responses (***p < 0.001) and higher reinforced responses for cocaine (+++p < 0.001 vs. saline) (n = 21 saline-veh, 21 nicotine-veh, 23 fluoxetine-veh). Enhancements of cocaine self-administration are no longer present after **(B)** acute 5-HT_1A_ receptor antagonism with WAY-100,635 (100 µg/kg, i.v.) (n = 8 saline-WAY, 7 nicotine-WAY, 8 fluoxetine-WAY) or **(C)** acute D_2_ receptor antagonism with L-741,626 (2 mg/kg, i.p.) (n = 12 saline-L-741626, 11 nicotine- L-741626, 11 fluoxetine- L-741626). Data were analyzed by 2-way ANOVA, with repeated measures on Responses. Significant effects of reinforcement were analyzed by Bonferroni-corrected paired t-tests for each pretreatment group. Bars represent mean ± SEM.

Following nicotine or fluoxetine pretreatment, enhanced acquisition of cocaine self-administration is seen in adolescent rats, with a significant effect of Pretreatment (F2,62 = 5.748, p < 0.01), Response (F1,62 = 47.807, p < 0.001), and a Response x Pretreatment interaction (F2,62 = 10.882, p < 0.001). Both nicotine and fluoxetine pretreatment significantly increased reinforced responses and preference for the reinforced hole compared to saline-pretreated controls (p < 0.001; **Figure 4A**). These pretreatment effects on acquisition of cocaine self-administration were no longer present following acute blockade of 5-HT_1A_ receptors with WAY-100,635 (**Figure 4B**) or acute blockade of D_2_ receptors with L-741,626 (**Figure 4C**). In contrast, pretreatment had no effect on initial sucrose self-administration in adolescents (**Supplementary Figure 4**).

### 3.4 Nicotine and fluoxetine alter regional 5-HT_1A_R activity in adolescent rats

Given behavioral evidence of changes in 5-HT_1A_ receptor activity following pretreatment with fluoxetine or nicotine in adolescents, we used a neuroanatomical approach to assess the regional specificity of these functional effects (**Supplementary Table 1**). With both pretreatments, drug- and region-specific changes were observed in subsequent acute stimulation of [^35^S]GTPγS binding to G proteins by the 5-HT_1A_ receptor agonist, 8-OH-DPAT. In the infralimbic cortex (IL), adolescent fluoxetine pretreatment enhanced 5-HT_1A_ receptor activity, with a significant overall effect of Pretreatment [F_2,52 = _3.754, p<0.05] and an Age × Pretreatment interaction [F_2,52 = _4.630, p<0.05]. *Post hoc* analysis showed that adolescent rats pretreated with fluoxetine had significantly greater 8-OH-DPAT stimulated [^35^S]GTPγS binding in the IL compared to saline-pretreated rats (p<0.01; **Table 1**). These effects were not seen in adolescents pretreated with nicotine or in adults.

**Table 1:** Nicotine and fluoxetine age-specifically and region-specifically alters 5-HT_1A_R activity in the infralimbic cortex (IL), medial amygdala (MeA), and primary motor cortex (M1).

		Adolescent			Adult		
		Saline	Nicotine	Fluoxetine	Saline	Nicotine	Fluoxetine
Motor Cortex	M1	0.80 ± 6.19	47.80 ± 12.21*	46.48 ± 19.87*	47.02 ± 22.66	13.80 ± 7.88	22.60 ± 6.91
	M2	21.42 ± 10.83	41.91 ± 8.30	54.92 ± 18.54	44.38 ± 10.28	29.33 ± 9.87	32.84 ± 7.67
Prefrontal Cortex	Cg1	19.66 ± 8.47	65.26 ± 13.06	71.83 ± 21.65	46.94 ± 13.02	14.88 ± 6.99	45.06 ± 15.93
	PrL	26.88 ± 11.00	41.47 ± 4.56	47.70 ± 16.70	53.37 ± 14.77	31.18 ± 6.70	30.37 ± 5.83
	IL	20.31 ± 7.02	40.59 ± 7.83	73.97 ± 17.97**	38.53 ± 7.51	21.35 ± 4.30	33.30 ± 7.55
	AI	8.17 ± 5.57	24.07 ± 4.63	27.74 ± 11.39	30.34 ± 7.63	23.91 ± 5.60	16.19 ± 4.61
	VLO	9.63 ± 4.56	11.45 ± 6.13	39.32 ± 11.95	19.86 ± 5.52	11.39 ± 6.65	16.87 ± 5.35
Amygdala	BLA	7.87 ± 5.43	5.69 ± 4.22	6.82 ± 4.14	11.81 ± 3.92	9.01 ± 4.61	-5.92 ± 6.62
	CeA	3.49 ± 4.66	3.97 ± 3.90	3.28 ± 5.14	9.74 ± 4.48	2.40 ± 3.23	-13.03 ± 5.61
	MeA	7.79 ± 5.57	32.06 ± 9.49*	9.26 ± 4.23	9.57 ± 2.32	11.54 ± 3.35	-2.81 ± 2.93

In the IL, adolescent fluoxetine pretreatment (1 mg/kg, i.v.) induced greater 8-OH-DPAT stimulated [^35^S]GTPγS binding compared to saline controls in the IL (* p ≤ 0.05, ** p < 0.01 vs. saline). In the MeA, adolescents nicotine pretreatment (60 ug/kg, i.v.) induced greater 8-OH-DPAT stimulated [^35^S]GTPγS binding compared to controls (* p < 0.05 vs. saline). In the M1, adolescents nicotine (60 µg/kg, i.v.) or fluoxetine (1 mg/kg, i.v.) pretreatment induced greater 8-OH-DPAT stimulated [^35^S]GTPγS binding compared to controls (* p < 0.05 vs. saline pretreatment). Across all adult regions, no significant effects of pretreatment on 8-OH-DPAT stimulated [^35^S]GTPγS binding were observed. Data were analyzed separately using 2-way ANOVA, with Dunnett’s *post hoc* tests. Data represent mean + SEM. n = 9–11/group.

Adolescent nicotine pretreatment enhanced 5-HT_1A_ receptor activity in the medial amygdala, with significant overall effects of Pretreatment [F_2,55 = _6.389, p<0.01] and Age [F_1,55 = _5.547, p<0.05]. *Post hoc* analysis showed that adolescent rats pretreated with nicotine had significantly greater 8-OH-DPAT stimulated [^35^S]GTPγS binding in this region compared to saline-pretreated rats (p<0.05; **Table 1**). These effects were not seen in adolescents pretreated with fluoxetine or in adults.

The primary motor cortex was the only region to show significant enhancement of 5-HT_1A_ receptor activity following adolescent pretreatment with either fluoxetine or nicotine, with a significant overall Age × Pretreatment interaction [F_2,54 = _4.919, p<0.05]. *Post hoc* analysis showed that both nicotine- and fluoxetine-pretreated adolescent rats had significantly higher 8-OH-DPAT stimulated [^35^S]GTPγS binding than saline-pretreated controls (p<0.05; **Table 1**). Drug pretreatment had no significant effects on 5-HT_1A_ receptor activity in adult.

## 4 Discussion

Consistent with a growing literature (3, 20, 40), we have shown that adolescents and adults respond quite differently to commonly used drugs, including nicotine and the SSRI, fluoxetine. Both drugs act via the 5-HT system to induce age-dependent changes in 5-HT_1A_R and D_2_ receptor activity. During adolescence, it is widely accepted that DA systems actively mature, whereas 5-HT systems exhibit comparatively low developmental activity since 5-HT neurons are among the first to mature during early brain development (41–43). In contrast, our present findings suggest that there are unique 5HT-DA interactions that occur during adolescence that influence the maturation of brain reward systems.

### 4.1 Serotonin signaling through 5-HT_1A_Rs regulates adolescent DA-mediated behaviors

Brief nicotine pretreatment during early adolescence has been shown to uniquely enhance DA-mediated behaviors, including cocaine self-administration, conditioned place preference, and locomotor sensitization as well as ambulatory activity induced by the D_2_ receptor agonist, quinpirole^,32–36^. We have previously shown that adolescent nicotine enhancement of quinpirole locomotion and cocaine self-administration is both mediated by 5-HT release and blocked by co-administration of selective 5-HT_1A_R antagonists (33). These findings are consistent with prior reports of unique activation of raphe by adolescent nicotine treatment (44). We now demonstrate that increased 5-HT activation of 5-HT_1A_Rs, resulting from fluoxetine blockade of transporter function, also increases quinpirole-induced locomotor activity and cocaine self-administration in adolescents but not adults. In the absence of a drug pretreatment, there are no age differences in the acquisition of cocaine self-administration (44), which indicates that endogenous 5-HT signaling regulates adolescent behavioral sensitivity to cocaine.

Treatment with 5-HT_1A_R antagonists during behavioral testing eliminates fluoxetine and nicotine pretreatment effects on quinpirole locomotion and cocaine reinforcement. This finding indicates that persistent enhancement of 5-HT_1A_R function mediates the behavioral effects of prior adolescent nicotine and fluoxetine exposure even after discontinuation of drug treatment. Since 5-HT_1A_Rs are located both pre- and post-synaptically (45), we compared the behavioral effects of two antagonists with differing pharmacological profiles to determine which 5-HT_1A_Rs mediated adolescent fluoxetine and nicotine effects. As an antagonist, WAY-100,635 is equally effective at pre- and post-synaptic 5-HT_1A_Rs. In contrast, S-15535 is a partial 5-HT_1A_R agonist, which acts functionally as an antagonist at postsynaptic receptors, but as an agonist at presynaptic autoreceptors (46). As with WAY-100635, acute administration of S-15535 immediately before behavioral testing blocked nicotine and fluoxetine enhancement of quinpirole-induced locomotion, indicating an involvement of postsynaptic 5-HT_1A_Rs.

Prior studies have shown that chronic fluoxetine leads to increased 5-HT system responsiveness after drug cessation, possibly because of 5-HT_1A_R autoreceptor desensitization (47). However, this effect is seen in both adolescents and adults, and does not result in changes in baseline monoamine levels. It is therefore unlikely to explain the increased postsynaptic 5-HT_1A_R sensitivity that we have observed following subchronic fluoxetine treatment in adolescents. Furthermore, using an *in vitro* approach to examine receptor-G protein coupling, we have shown unique effects of adolescent fluoxetine and nicotine exposure on 5-HT_1A_R function.

Fluoxetine enhanced 5-HT_1A_R activity in the IL, a prefrontal cortical region important for emotional learning and habit behavior (48, 49). 5-HT_1A_Rs in this region have been implicated in modulating emotional response to stressful situations (50). In contrast, nicotine enhanced 5-HT_1A_R activity in the medial amygdala, a region of the amygdala implicated in control of sexual and social behaviors (51, 52). 5-HT_1A_Rs in this region have been implicated in innate fear modulation (53). However, both fluoxetine and nicotine both enhanced 5-HT_1A_R activity only in the M1, a cortical motor region that experiences continuous growth in serotoninergic innervation until late adolescence (54). The M1 is actively engaged during initial motor consolidation and plays a critical role in acquisition of skill learning (55). 5-HT_1A_Rs within M1 decrease corticostriatal activity (56) and modulate DA-mediated motor function (57). Since both fluoxetine and nicotine enhanced 5-HT_1A_Rs activity within this region, it is possible that M1 may be the site of action of these drugs in enhancing adolescent DA-mediated behaviors.

### 4.2 Adolescent fluoxetine and nicotine enhance function of D_2_ dopamine receptors

The induction of locomotor activity by quinpirole in adolescents is mediated by D_2_ receptors and is age-specific (36). As confirmed in the present study, adults do not show a locomotor response to quinpirole (33, 58), a finding that is consistent with the maturation of DA systems and D_2_ receptor function that occurs during adolescence (1, 3, 5). We have previously shown that adolescent nicotine pretreatment enhances quinpirole-induced locomotor activity via a 5-HT_1A_R mechanism and increases D_2_ receptor expression and functional activity in the striatum (32, 33). Involvement of the D_2_ receptor is further indicated by the finding that D2 receptor blockade or knockdown during nicotine pretreatment of adolescents blocks subsequent enhancement of cocaine self-administration via a microglia-specific mechanism (33). We now show that fluoxetine pretreatment enhances adolescent locomotor response to quinpirole, indicating that increased serotonergic signaling during pretreatment potentiates subsequent D_2_ receptor function in this age group. Adolescent nicotine and fluoxetine enhancement of cocaine self-administration is also mediated by increased functional activity of D_2_ receptors, since the effect is blocked by treatment with a D_2_-specific antagonist, L-741,626, during self-administration testing. The finding that cocaine self-administration in adolescents is mediated by D_2_ receptors is in marked contrast to adults, where D_1_ receptors mediate cocaine reinforcement (59). Indeed, D_2_ receptors do not mediate cocaine self-administration in adults but are, instead, involved in mechanisms that limit intake of high-dose cocaine (60).

As shown in the present study, fluoxetine and nicotine enhancement of DA-mediated behaviors in adolescents are mediated by functional sensitization of both 5-HT_1A_R and D_2_ receptors. Enhanced behavioral response following pretreatment with either drug is eliminated by administration of either 5-HT_1A_R or D_2_ receptor antagonists immediately prior to testing. This suggests that there is a close interaction of 5-HT_1A_R and D_2_ receptors in adolescent brain that modulates reward circuitry. The mechanisms underlying such coupling of receptor activity are presently unknown but may either involve direct receptor interaction or a circuit based mechanism. Further studies will be required to evaluate the exact nature of this linked receptor response.

### 4.3 Limitations

The findings in this report are subject to at least three limitations. First, this study was conducted in males since we have previously shown that adolescent nicotine enhancement of cocaine self-administration is seen in both males and females (32, 33). However, further studies will be required to determine whether this is also the case for fluoxetine in females. Second, pretreatment with nicotine does not fully capture the myriad of other psychoactive constituents that are present in tobacco leaves, combustible tobacco products, or e-cigarette vaping liquids, and further investigations are needed in order to characterize how these other psychoactive constituents interact with nicotine and fluoxetine. Finally, since adolescent and adult rats received pretreatment for 4 consecutive days and behavioral testing was conducted the following day, our study is limited to the short-term effects of drug exposure and acute pharmacological interventions. Additional studies exploring the long-term consequences of drug exposure during adolescence could better inform the effectiveness of pharmacological interventions on behavior later in adulthood.

### 4.4 Clinical Implications

With increasing evidence that aberrant activation of 5-HT systems during adolescence triggers lasting changes in neuronal signaling, use of drugs such as tobacco, e-cigarettes, and antidepressants may permanently alter the course of adolescent brain maturation and neurotransmitter signaling. Although teen smoking is declining, teen vaping has increased greatly in recent years (6, 7) and has been suggested to be a gateway to future tobacco use (61, 62). We have now shown that nicotine disrupts normative limbic development and sensitizes the adolescent brain to DA drugs such as cocaine. Thus, as with tobacco, e-cigarettes may prime behavioral susceptibility to drugs of abuse in teenagers with lifelong consequences for mental health.

Treatments for mental health disorders in teens also pose serious safety concerns. Even though fluoxetine is FDA approved to treat teen depression, the majority of testing regarding its effectiveness and safety has been conducted in adults (63, 64). In addition, fluoxetine’s antidepressant effects require chronic administration in order to be therapeutic, suggesting postsynaptic neuroadaptations in 5-HT receptor signaling (65). However, we have demonstrated that changes in adolescent behavior after nicotine or fluoxetine exposure can be blocked with an acute treatment of a 5-HT_1A_R antagonist or partial agonist.

Prior studies have shown that 5-HT_1A_R antagonists are safe for clinical use. The delayed antidepressant effects of SSRIs can be reduced by co-treatment of pindolol, a nonselective beta blocker with 5-HT_1A_R antagonist activity (66, 67). The 5-HT_1A_R partial agonist busipirone – which has long been used to treat anxiety – also demonstrates clinical efficacy when given in combination with SSRIs (68). Recently, vilazodone, which has combined SSRI and 5-HT_1A_R partial agonist properties, has been approved for treatment of major depressive disorder in adults (69). Since we have shown that 5-HT_1A_R antagonists and partial agonists attenuate adverse behavioral responses to antidepressants in adolescents, treatment for teen depression should take into consideration potential drug therapies with 5-HT_1A_R antagonist activity to improve antidepressant efficacy, particularly during the initial period when adolescents are most vulnerable to the confounding effects of SSRI treatment. Furthermore, this body of work indicates that 5-HT_1A_Rs are also viable drug targets for the treatment of adverse health consequences after adolescent exposure to tobacco, e-cigarettes, and antidepressants.

## Data availability statement

The raw data supporting the conclusions of this article will be made available by the authors, without undue reservation.

## Ethics statement

The animal study was approved by Institutional Animal Care and Use Committee (IACUC). The study was conducted in accordance with the local legislation and institutional requirements.

## Author contributions

MY: Conceptualization, Formal analysis, Methodology, Project administration, Visualization, Writing – original draft, Writing – review & editing. FL: Conceptualization, Funding acquisition, Methodology, Resources, Supervision, Writing – original draft, Writing – review & editing.

## Supplementary material

Supplementary Table 1 Regional 5-HT1A receptor activity after nicotine or fluoxetine pretreatment in adolescent and adult brain slices. [35S]GTPγS binding is expressed (Mean +/- SEM) as % of basal. Asterisks (*) denote significant differences from saline. n = 9-11/ group.

Supplementary Figure 1 Experimental timeline. Adolescents are represented in red, and adults are in black.

Supplementary Figure 2 Representative autoradiograms for 8-OH-DPAT-stimulated [35S]GTPγS binding. Sample images were taken from adult (P90) brain slices after saline pretreatment.

Supplementary Figure 3 Nicotine and fluoxetine pretreatments did not alter cocaine self-administration during adulthood. There were no significant differences in responses between pretreatment groups during cocaine self-administration in adult rats. n = 8-10/group.

Supplementary Figure 4 Nicotine and fluoxetine pretreatments did not alter sucrose self-administration during adolescence. There were no significant differences in responses between pretreatment groups during sucrose self-administration in adolescent rats. n = 6/group.
