# Supplementary figures and images for: Nicotine and fluoxetine alter adolescent dopamine-mediated behaviors via 5-HT1A receptor activation
Source: Front Psychiatry. 2024 Jun 11;15:1380123. doi: 10.3389/fpsyt.2024.1380123 (PMC11196788; doi:10.3389/fpsyt.2024.1380123)

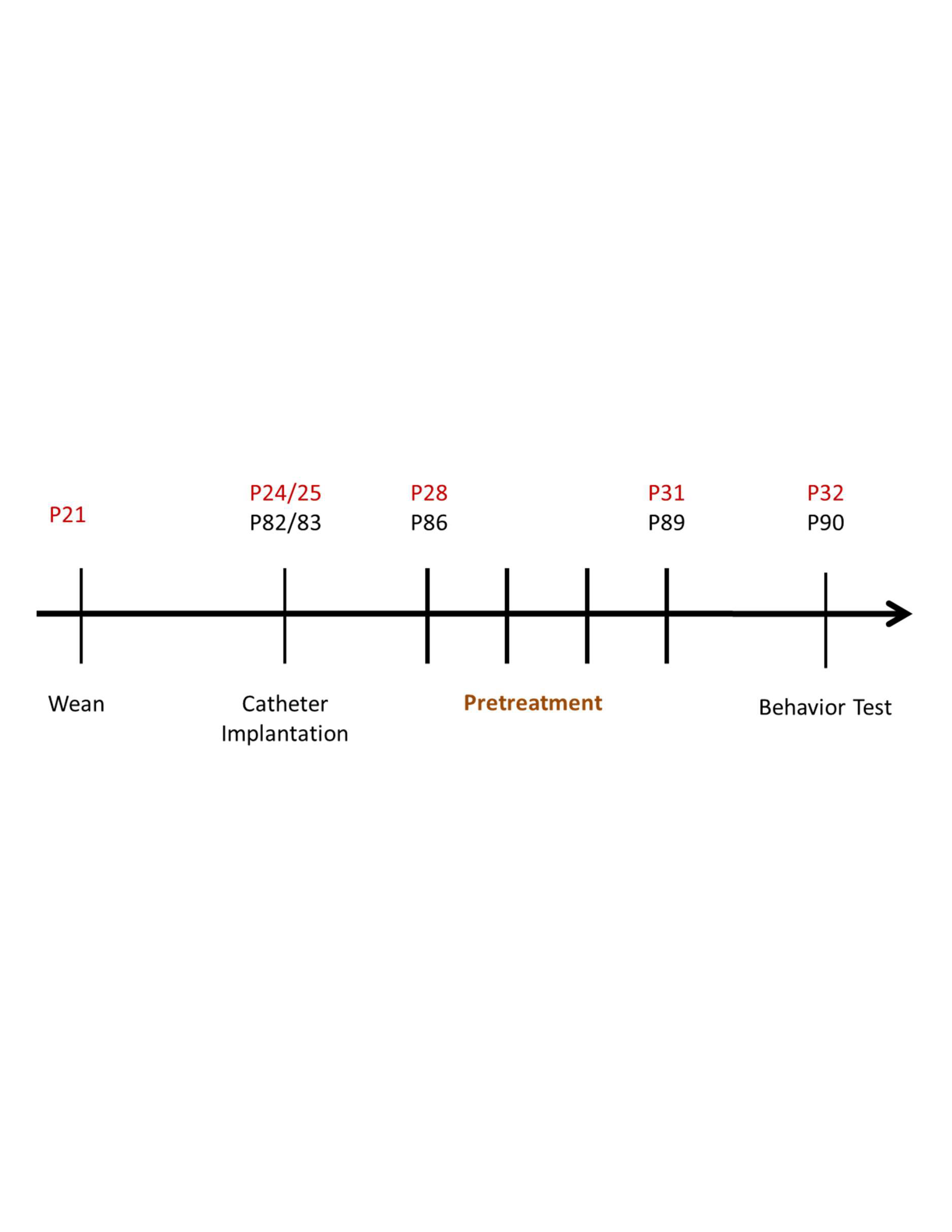

Supplement: Supplementary Figure 1 — Experimental timeline. Adolescents are represented in red, and adults are in black. [file Image_1.tiff]

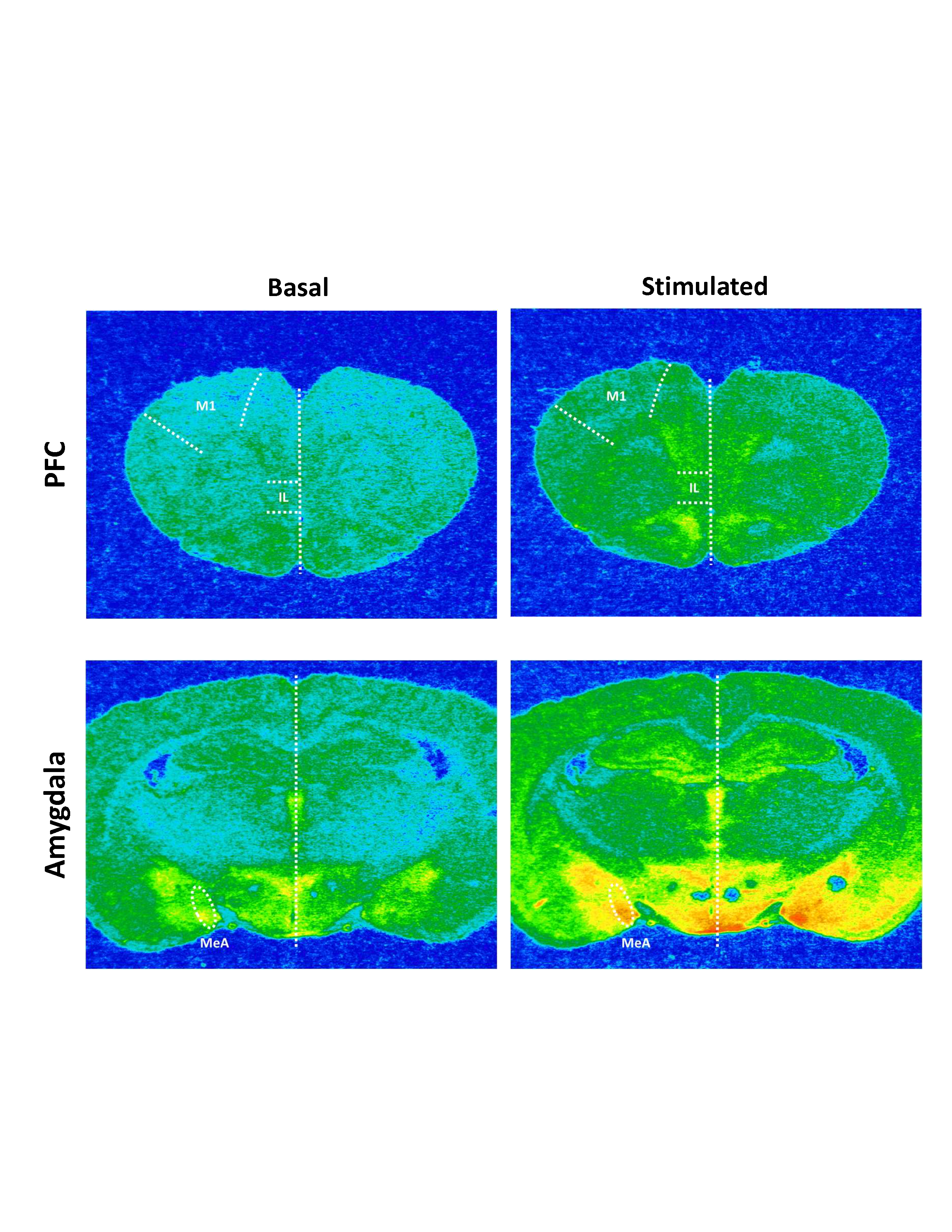

Supplement: Supplementary Figure 2 — Representative autoradiograms for 8-OH-DPAT-stimulated [35S]GTPγS binding. Sample images were taken from adult (P90) brain slices after saline pretreatment. [file Image_2.tiff]

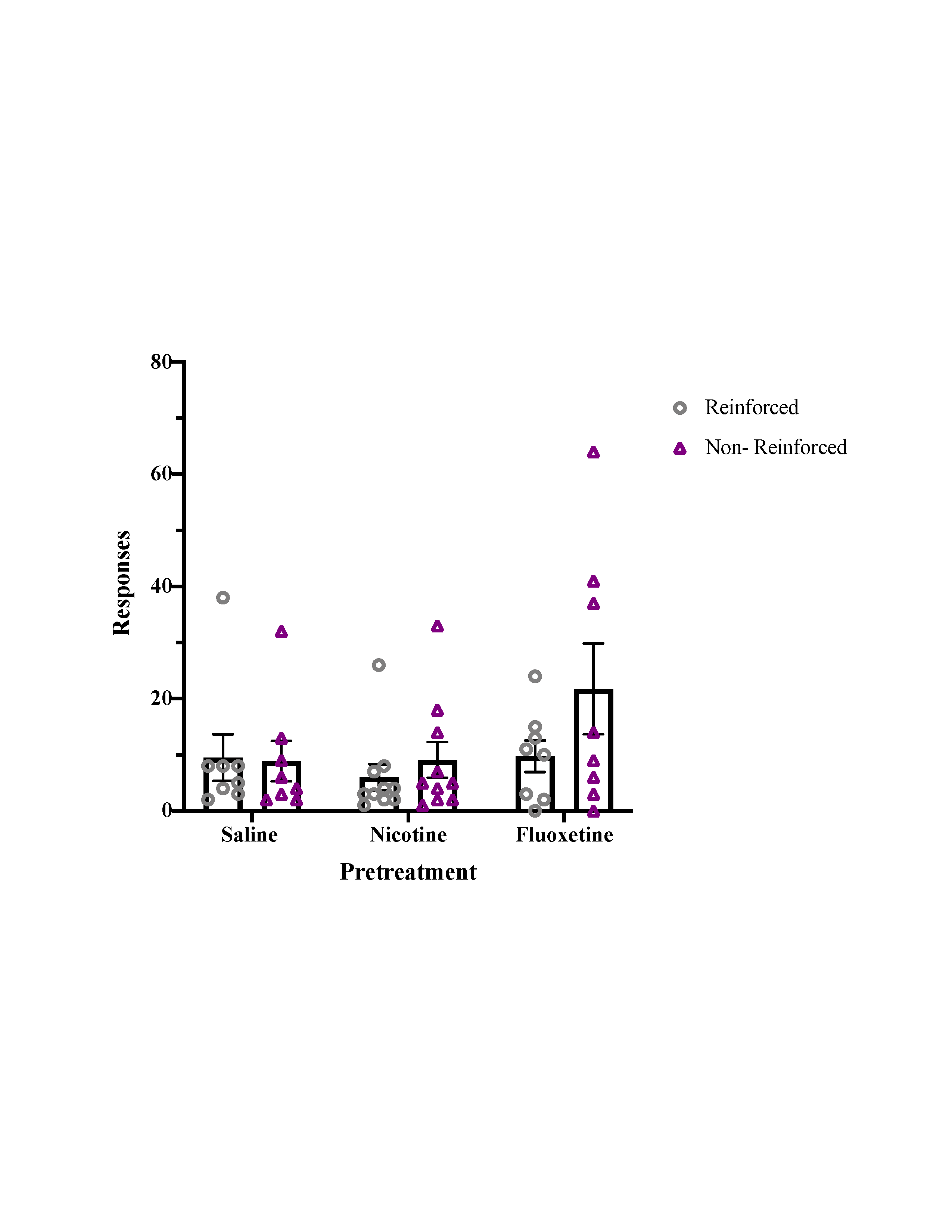

Supplement: Supplementary Figure 3 — Nicotine and fluoxetine pretreatments did not alter cocaine self-administration during adulthood. There were no significant differences in responses between pretreatment groups during cocaine self-administration in adult rats. n = 8-10/group. [file Image_3.tiff]

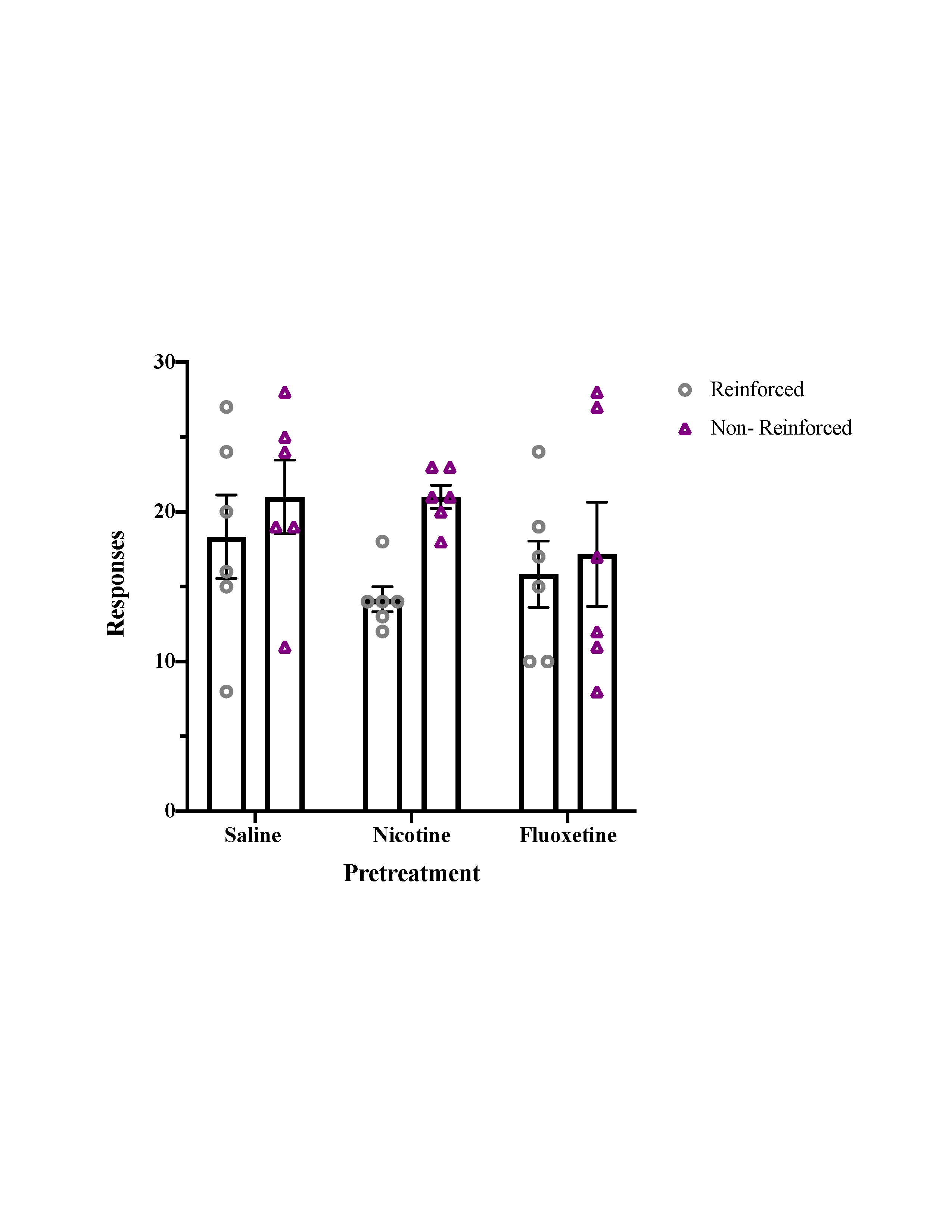

Supplement: Supplementary Figure 4 — Nicotine and fluoxetine pretreatments did not alter sucrose self-administration during adolescence. There were no significant differences in responses between pretreatment groups during sucrose self-administration in adolescent rats. n = 6/group. [file Image_4.tiff]
